# Microsatellite Diversity, Complexity, and Host Range of Mycobacteriophage Genomes of the *Siphoviridae* Family

**DOI:** 10.3389/fgene.2019.00207

**Published:** 2019-03-14

**Authors:** Chaudhary Mashhood Alam, Asif Iqbal, Anjana Sharma, Alan H. Schulman, Safdar Ali

**Affiliations:** ^1^Luke/BI Plant Genome Dynamics Lab, Institute of Biotechnology and Viikki Plant Science Centre, University of Helsinki, Helsinki, Finland; ^2^Ingenious e-Brain Solutions, Gurugram, India; ^3^PIRO Technologies Private Limited, New Delhi, India; ^4^Department of Biomedical Sciences, SRCASW, University of Delhi, New Delhi, India; ^5^Natural Resources Institute Finland (Luke), Helsinki, Finland; ^6^Department of Biological Sciences, Aliah University, Kolkata, India

**Keywords:** Mycobacteriophage, simple sequence repeats, imperfect microsatellite extractor, dMAX, host range

## Abstract

The incidence, distribution, and variation of simple sequence repeats (SSRs) in viruses is instrumental in understanding the functional and evolutionary aspects of repeat sequences. Full-length genome sequences retrieved from NCBI were used for extraction and analysis of repeat sequences using IMEx software. We have also developed two MATLAB-based tools for extraction of gene locations from GenBank in tabular format and simulation of this data with SSR incidence data. Present study encompassing 147 Mycobacteriophage genomes revealed 25,284 SSRs and 1,127 compound SSRs (cSSRs) through IMEx. Mono- to hexa-nucleotide motifs were present. The SSR count per genome ranged from 78 (M100) to 342 (M58) while cSSRs incidence ranged from 1 (M138) to 17 (M28, M73). Though cSSRs were present in all the genomes, their frequency and SSR to cSSR conversion percentage varied from 1.08 (M138 with 93 SSRs) to 8.33 (M116 with 96 SSRs). In terms of localization, the SSRs were predominantly localized to coding regions (∼78%). Interestingly, genomes of around 50 kb contained a similar number of SSRs/cSSRs to that in a 110 kb genome, suggesting functional relevance for SSRs which was substantiated by variation in motif constitution between species with different host range. The three species with broad host range (M97, M100, M116) have around 90% of their mono-nucleotide repeat motifs composed of G or C and only M16 has both A and T mononucleotide motifs. Around 20% of the di-nucleotide repeat motifs in the genomes exhibiting a broad host range were CT/TC, which were either absent or represented to a much lesser extent in the other genomes.

## Introduction

Phages are the most abundant organisms in the biosphere; the entire population turns over every few days (Brüssow and Hendrix, 2002; Comeau et al., 2008; Hatfull, 2015). Diversity of the bacteriophage population at the genetic level is highly dynamic due to horizontal exchange of segments between genomes (Wommack and Colwell, 2000; Hendrix, 2004; Suttle, 2007). Furthermore, virion structures suggest that bacteriophages are extremely old (Krupovič and Bamford, 2010). In spite of their age, diversity and ubiquity, bacteriophage comparative genomics has not attracted as much attention relative as has that of other microbial genomes. One reason may be the lack of individual isolates for genomic analyses (Hatfull and Hendrix, 2011). However, due to advancements in sequencing and analysis techniques, over 2000 completely sequenced bacteriophage genomes are now available in the GenBank database^1^.

Important feature of the genomes, which have been extensively served as a tools for comparative and evolutionary genomics, are the SSRs. The SSRs are tandem repetitions of relatively short DNA motifs present in interrupted; pure; compound; interrupted-compound; complex or interrupted-complex forms (Table 1; Chambers and MacAvoy, 2000). They are present in diverse taxa across viruses, prokaryotes, and eukaryotes (Gur-Arie et al., 2000; Kofler et al., 2008; Chen et al., 2012). Functionally, these sequences are associated with gene regulation, transcription, and protein function in prokaryotes and eukaryotes (Kashi and King, 2006; Usdin, 2008), though the presence and role of SSR in viruses (Ouyang et al., 2012) remains to be exhaustively studied. Genome features including size and GC content influence the occurrence and complexity of SSRs (Dieringer and Schlötterer, 2003; Coenye and Vandamme, 2005; Kelkar et al., 2008). However, this correlation is not universal and therefore a single rule for their incidence cannot be forged.

**Table 1:** Types of micrptosatellites present in the study.

S. No.	Class	Sequence	Source
1	Pure	-(CG)_3_-	Supplementary Table 1 (M1)
2	Interrupted pure	-(CA)_3_-x_9_-(CA)_3_-	Supplementary Table 2 (M9)
3	Compound	-(GT)_5_-x_0_-(CG)_3_-	Supplementary Table 2 (M1)
4	Interrupted compound	-(GA)_3_-x_8_-(AAG)_9_-	Supplementary Table 2 (M1)
5	Complex	-(CG)_3_-x_7_-(GC)_3_-x_1_-(CG)_3_-	Supplementary Table 2 (M1)
6	Interrupted complex	-Complex-X_5_-Complex-	Not available in the analysis

Phages replicates through lytic and lysogenic cycles depending on the benefit of being virulent versus temperate. In M13 bacteriophages, it has been reported that two-triplet repeats can impede DNA replication not by means of hairpin structures, but due to increased free energy on interaction with flanking DNA sequences and unique secondary structure (Pan, 2004). Till now there is no report of any repeat sequence present in specific protein of phages which is responsible for function of that particular protein. Microsatellites have been extensively used for DNA fingerprinting, forensics and evolution studies (Queller et al., 1993); however, their role in genomes as a whole remains to be ascertained. Here, we focus on viruses belonging to 15 Genera of Mycobacteriophages as an attempt to understand the evolution of microsatellites and their host viral genomes.

## Materials and Methods

### Genome Sequences

Complete genome sequences of 147 Mycobacteriophages from 15 genomes were retrieved from NCBI^2^ and analyzed for simple and compound SSRs (cSSRs). These included the following genera, the numbers in parentheses representing the number of species in the genus: *Barnyardlikevirus* (4), *Bignuzlikevirus* (2), *Bronlikevirus* (4), *Charlielikevirus* (2), *Che8likevirus* (28), *Che9clikevirus* (3), *Cjwunalikevirus* (9), *Corndoglikevirus* (2), *Halolikevirus* (2), *Omegalikevirus* (6), *Pbiunalikevirus* (1), *Pgonelikevirus* (12), *Reylikevirus* (2), *Tm4likevirus* (9), and *L5likevirus* (61). The species included in the study and their genome features have been summarized in Supplementary Table 1.

### Microsatellite Extraction

Searches for microsatellites were carried out using the “Advance-Mode” of IMEx with the parameters reported for HIV (Mudunuri and Nagarajaram, 2007; Chen et al., 2012): Type of Repeat, perfect; Repeat Size, all; Minimum Repeat Number 6(mono-), 3(di-), 3(tri-), 3(tetra), 3(penta-), 3(hexa); Maximum distance allowed between any two SSRs (dMAX), 10. Two SSRs separated by a distance of less than 10 bp were thus treated as a single cSSR. Other parameters were set to the defaults.

### Statistical Analysis

Microsoft Office Excel 2007 was used to perform all statistical analyses. Linear regression was used to reveal the correlation between the RA, RD of microsatellites with genome size.

Compound microsatellite statistical significance in each genome was assessed using *Z*-scores which is defined as (O–E)/√E (Mrázek, 2006). *Z*-scores were calculated using equations:

$$\begin{array}{l} c^{\prime }=1/n\sum_{i=1}^{n}\left(\frac{CcSSRi}{cSSRi}\right)\\ cSSRexp=CcSSR/c^{\prime }\\ z=O-E/\sqrt{E} \end{array}$$

Where count of individual microsatellite being part of compound microsatellite is represented by CcSSR, “O” is the cSSR observed in each genome (cSSR_obs_) and “E” is the cSSR expected in each genome (cSSR_exp_).

GraphPad prism 7 was used to do Chi-square test. Restricted host range and broad host range were chosen as two category and different repeat sequence were taken as groups.

### MATLAB-Based Tools for SSR Analysis

Imperfect microsatellite extractors (IMEx) is widely used to find SSRs in genomes. However, connecting these SSRs to associated gene has remained a manual process. In order to expedite the process, we have developed two MATLAB-based tools: Identification of Gene Location from NCBI Nucleotide File (IGLNNF) and Incorporation of Gene Location in SSR File (IGLSF). IGLNNF obtains gene locations from GenBank directly and saves them into.xlsx format. IGLNNF requires two inputs: accession number (of the sequence to be analyzed); filename (where extracted gene locations will be stored). The IGLNNF tool will be made available for research and teaching purposes at our website: www.pirotechnologies.com/cmdownloads/identification-of-gene-location-from-ncbi-nucleotide-file/.

IGLSF incorporates the gene location into the SSR file. IGLSF requires two inputs: gene file; SSR file, into which locations will be incorporated. Both the inputs must be in.xlsx format and can be uploaded by clicking on “Upload Gene File” and “Upload SSR File” respectively. The output can be obtained by clicking on Simulate button. The IGLSF tool will be available for research and teaching purposes at our website:

www.pirotechnologies.com/cmdownloads/incorporation-of-gene-location-in-SSR-file/.

### Dot Plot Analysis and Host Range

Dot matrix analysis is used to compare two nucleic acid or protein sequences. Dot plots for representative genomes was developed using Genome Pair Rapid Dotter (GEPARD) (Krumsiek et al., 2007) to highlight the presence of SSRs within the genomes. The graphical results of dot matrix analysis is known as dot plot which is used to examine the evolutionary relationships of the sequences by analyzing repeats, reverse matches, and conserved domains.

Pearson Chi-squared test was performed to ascertain the significance of SSR motif distribution with reference to host range as in to check whether the observations were a chance occurrence as per standard protocols (Pearson, 1900; Oliveira et al., 2018).

## Results

### Occurrence of SSRs and cSSRs

**FIGURE 1:**
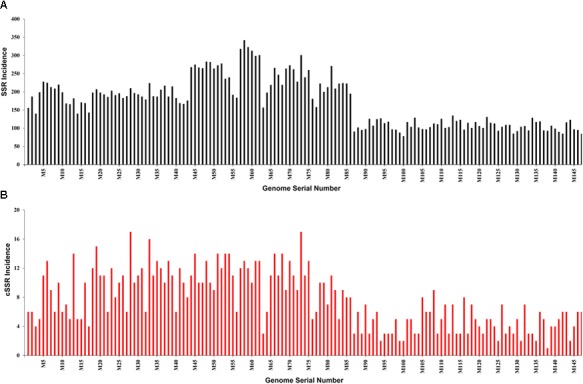
**(A)** Incidence of SSRs and **(B)** cSSRs in the studied Mycobacteriophage genomes. Note the highest SSR and cSSR incidence of 342 (M58) and 78 (M100) whereas corresponding values for cSSR are 17 (M28) and 1 (M138) respectively.

**FIGURE 2:**
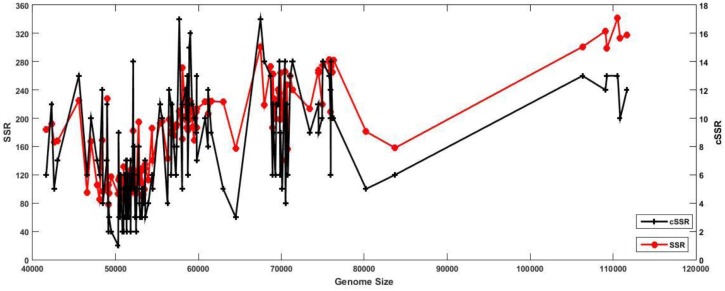
Relation between genome size and SSR/cSSR incidence. The presence of some of the highest peaks (SSR/cSSR incidence) on the far left of the *X*-axis (smaller genome size) are a clear indication of comparable SSR incidences across varying length of genomes, thus implying their functional significance.

Genome-wide searches for microsatellites across 147 mycobacteriophage genomes revealed 25,284 SSRs and 1,127 cSSRs (Supplementary Tables 1–3). The SSR count per genome ranged from 78 (M100 – *Mycobacterium phage* *D29*) to 342 (M58 – *Mycobacterium phage courthouse*) (Figure 1A). The genome sizes in the studied species ranges from 41,650 to 111,688 bp, while the GC content ranges from 50.3 (M3) to 69.1 (M66, M67) (Supplementary Table 1). The genome size range can account for variation in the SSR incidence in principle: To uncover the actual scenario, we plotted genome size with SSR and cSSR incidence. As evident from the Figure 2, there are multiple examples of smaller genomes with disproportionately many SSRs and vice versa. For instance, the smallest genome, that of M56 (41,650 bp) has 184 SSRs, which more than twice the least number of SSRs present in another genome (M100, 49,136 bp). Also, M76, with 181 SSRs, a number similar to that in M46, has a much larger genome at 80,228 bp (Figure 2).

**FIGURE 3:**
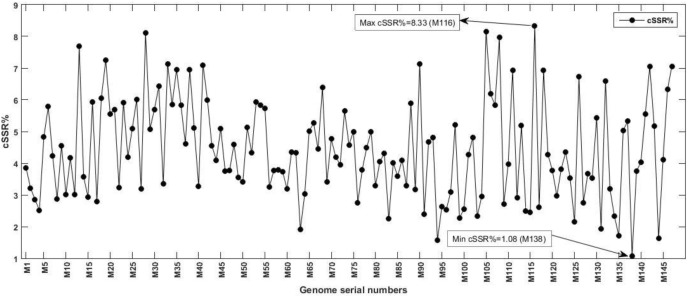
Compound SSRs % in the studied Mycobacteriophage genomes. The percentage of individual microsatellites that are part of a compound microsatellite is represented by cSSR %. Note the presence of highest cSSR% of 8.33 in M116 with just 96 SSRs (Supplementary Table 1), representing uneven distribution of SSRs, suggestive of functional relevance.

The incidence of cSSRs ranged from 1 (M138) to 17 (M28, M73) (Figure 1B and Supplementary Tables 1–3). The analyses reveal that a higher incidence of SSR doesn’t necessarily correlate with a higher number of cSSRs. For instance, *Mycobacterium phage tiger* (M138), with 93 SSRs, has a single cSSR, whereas *Mycobacterium phage L5* (M116), with 96 SSRs has 8 cSSRs (Figure 1 and Supplementary Tables 1–3). Furthermore, we looked into cSSR percentage, which is the percentage of SSRs in a genome present as cSSRs (Figure 3); this ranged from 1.08 (M138 with 93 SSRs) to 8.33 (M116 with 96 SSRs) (Supplementary Table 1).

### Relative Abundance and Relative Density of SSRs and cSSRs

**FIGURE 4:**
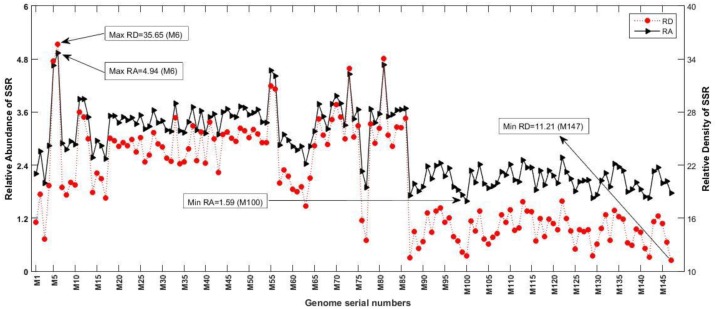
Relative abundance (RA) and relative density (RD) of SSRs. RA is the number of microsatellites present per kb of the genome whereas RD is the sequence space composed of SSRs of microsatellites per kb of the genome. The variations in these variables represent incidence and distribution of these sequences across genomes.

**FIGURE 5:**
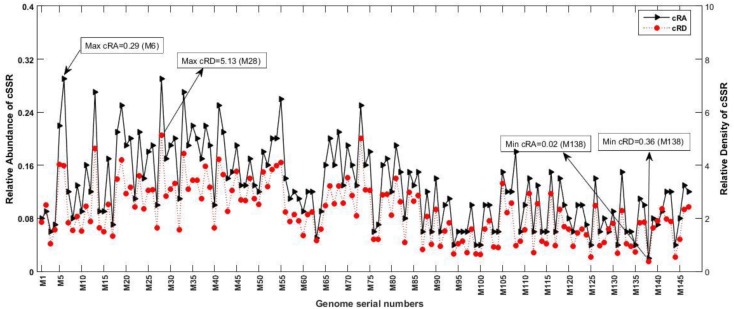
Relative abundance (cRA) and relative density (cRD) of cSSRs. cRA is the number of compound microsatellites present per kb of the genome whereas cRD is the sequence space composed of cSSRs per kb of the genome.

Owing to the variable SSR and cSSR frequencies, we looked into their RA and RD. The RA is the number of microsatellites present per kb of the genome whereas RD is the sequence space composed of SSRs per kb of the genome. The RA of SSRs ranged from “1.59 (M100) to 4.94 (M6)” and for cSSRs from “0.02 (M138) to 0.29 (M6)” (Supplementary Table 1 and Figure 4, 5). The RD of SSRs ranged from “11.21 (M147) to 35.65 (M6)” and for cSSR from “0.36 (M138) to 5.13 (M28)” (Supplementary Table 1 and Figure 4, 5). The count of SSR in compound microsatellite (cSSR) ranged from 36 in M73 (*Mycobacterium phage rosebush*) to 2 in M138 (*Mycobacterium phage tiger*). The cSSR%, which is percentage of individual microsatellite being part of compound microsatellite was (18.4%) highest for M105 (*Mycobacterium phage gladiator*) and (2.2%) lowest for M138 (Supplementary Table 1).

### SSR Motif Types, Iterations, and cSSR Complexity

**FIGURE 6:**
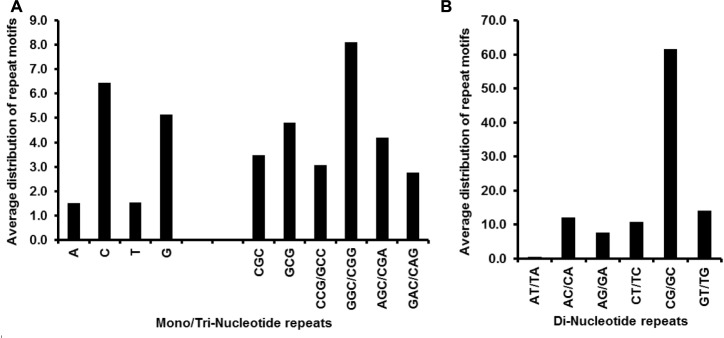
**(A)** Average distribution of mono- and tri-nucleotide repeat motifs and **(B)** di-nucleotide repeat motifs. The most prevalent mono-, di- and tri-nucleotide repeat motifs are “C”, “CG/GC,” and “GGC/CGG” respectively, which corroborates with the GC rich nature of the studied genomes.

We also looked into the divergence of repeat motifs extracted from the Mycobacteriophage genomes. The SSRs repeat motifs ranged from mononucleotides to hexanucleotides. Of the observed mononucleotides, the most prevalent one was a C repeat, with an average distribution of over six across the studied genomes, followed by T as shown in Figure 6A. The A and G mononucleotide motifs were least represented (average distribution 1.5 each). Among the dinucleotide repeats, the CG/GC repeat motif was the most prevalent with an average distribution of ∼62 across studied genomes, with GT/TG a distant second and having an average distribution of 13 (Figure 6B). The CGG/GGC motif was the highest incident trinucleotide repeat. The distribution of these repeats has been illustrated in Figure 6A. Regarding the number of iterations present at a stretch, a maximum of 12 repeats were present for mono-nucleotide C and G each in M129 and M139 respectively. The dinucleotide repeat motifs AC had the highest iteration of 9 observed in M2. The trinucleotides had a higher iteration than the dinucleotides and most of them are coding for amino acids. The AAG motif was present with 9 repeats in M1.

### dMAX and cSSR

**FIGURE 7:**
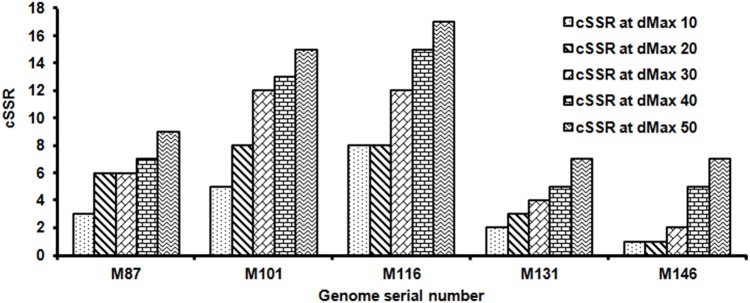
Frequency of cSSR in relation to varying dMAX (10–50) across five randomly selected Mycobacteriophage genomes. A higher cSSR incidence with increasing dMAX in the genomes is along expected lines but the non-linearity of the increase across species is suggestive of genome specific clustering of SSRs.

dMAX is defined as the maximum permissible distance between any two adjacent microsatellites for them to be classified as compound microsatellite (Kofler et al., 2008). The cSSRs described above have a dMAX value of 10. To determine the impact of varying dMAX on cSSR incidence, five genomes, M87, M101, M116, M131, and M146, were chosen at random and the cSSRs were extracted with increasing dMAX. The dMAX value can be set only between 0 and 50 for IMEx (Mudunuri and Nagarajaram, 2007). As expected, there was an increase in cSSR with higher dMAXs in the studied Mycobacteriophage genomes (Figure 7). However, the increase was neither linear nor uniformly proportional across species. For instance, there was no increase in cSSR percentage in M87, M116, or M146 when the dMAX increased from 20 to 30, 10 to 20, and 40 to 50 respectively (Figure 7). This variation is indicative of the differential distribution of SSRs; the motifs are much closely packed in M146 as compared to M116. This is significant as the ability of motifs to induce variations is often dependent on their proximity with other motifs.

### SSRs in Coding Regions

**FIGURE 8:**
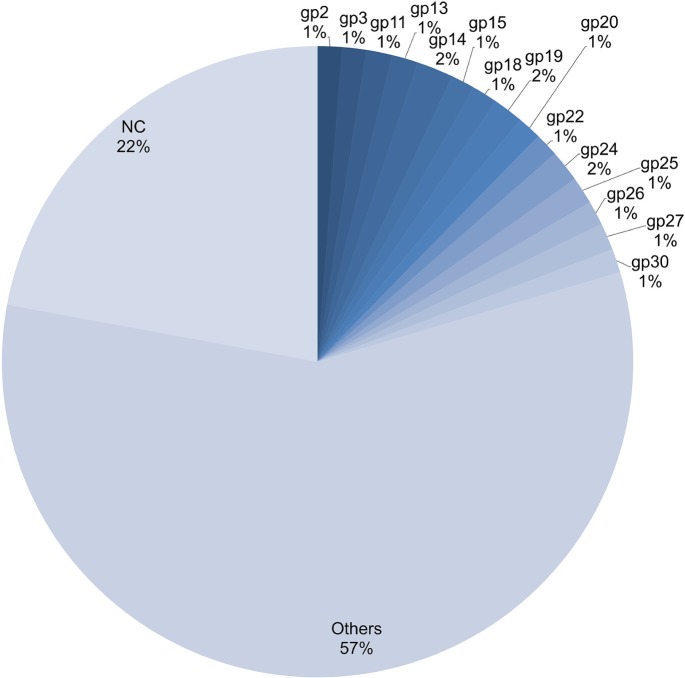
Differential distribution of SSRs (%) in coding vs. non-coding regions. In the figure “gp” represents “ORF”. The 15 most conserved “gp” were included in this figure, “NC” represents non-coding and “Others” represent in remaining “gp” (179). The numbers in percentage represent the fraction of SSRs that can be attributed to that specific sequence across genomes.

**FIGURE 9:**
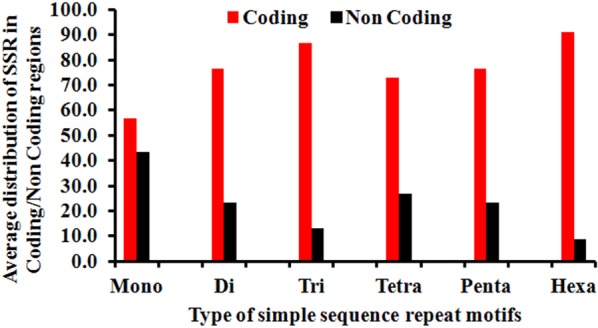
Differential distribution of individual SSR (%) from Mono to Hexanucleotide in coding vs. non-coding regions. The figure very clearly illustrates the extreme bias of hexanucleotide repeats incidence in coding regions. This was followed by trinucleotide repeats whereas the least bias was observed in case of mononucleitide repeats.

We also explored the distribution of SSRs across coding and non-coding regions of the genome. This was accomplished by first extracting the genome locations of genes or putative genes into excel format using IGLNNF. The CDS sequence represents the coding part of a gene. A total of 194 coding sequences (CDS) were thus obtained. Generally, these sequences have not been annotated or their function further studied. Subsequently, this data was simulated with the SSR data through IGLSF to get the distribution across coding and non-coding regions. The SSRs distribution across coding and non-coding region was approximately 78 and 22% respectively (Supplementary Table 2). For our analysis, we used 15 conserved CDS domains, which were present in the greatest number of species and studied the percentage of SSRs each of these CDS accounted for as summarized in Figure 8. Further, on looking at the types of SSR motifs present in coding and non-coding regions, we found that average density of hexanucleotides was greatest in coding regions, followed by that of trinucleotide repeats (Figure 9). Overall, the number of dinucleotide repeats (15710) was greatest, followed by trinucleotide repeat (7231) motifs. The rarest class of repeats present was the pentanucleotides (17 motifs).

### Correlation Studies

We tested for correlations between genome size and GC content and the number, RA, and RD of SSRs and cSSRs. Correlations between genome size and SSR parameters were significant in terms of incidence (*R*^2^ = 0.6, *P* < 0.05), RA (*R*^2^ = 0.06, *P* < 0.05) and RD (*R*^2^ = 0.05, *P* < 0.05). The similarly correlation was significant for GC content in terms of RA (*R*^2^ = 0.13, *P* < 0.05) and RD (*R*^2^ = 0.16, *P* < 0.05), but insignificant for incidence (*R*^2^ = 0.02, *P* > 0.05), Correlation analysis was performed as well for cSSRs. The correlation between genome size and cSSR parameters were insignificant for incidence (*R*^2^ = 0.2, *P* > 0.05), yet significant with cRA (*R*^2^ = 0.003, *P* < 0.05) and cRD (*R*^2^ = 0.01, *P* < 0.05). GC content was not significantly correlated with cSSR incidence (*R*^2^ = 0.03, *P* > 0.05) and cRD (*R*^2^ = 0.06, *P* > 0.05), but was significant for cRA (*R*^2^ = 0.07, *P* > 0.05). The *Z*-scores served to test the statistical significance of the compound microsatellite distribution in 96 species. cSSR_obs_ was higher compared to cSSR_exp_, its value ranging from 0 to 0.12, whereas in 51 species cSSR_obs_ was lower compared to cSSR_exp_ and ranged from -0.02 to -0.18, respectively.

### SSRs and Host Range

**FIGURE 10:**
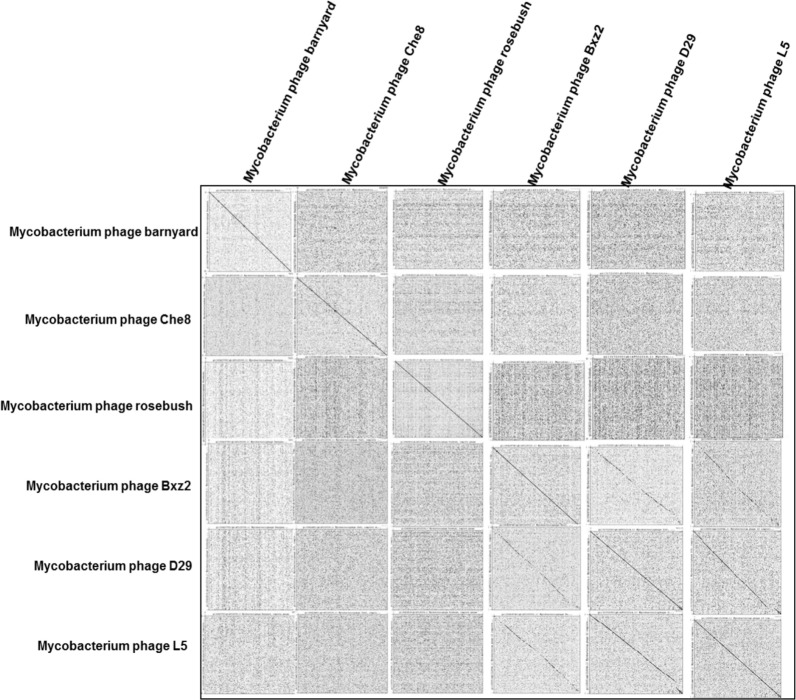
Dot plot analysis of six Mycobacteriophage genomes, three with broad host range and three with restricted host range. Repeats within a single genome are depicted as dots, which extend into lines with as the repeats extend. Lines off the center line of the global comparison indicate sequence conservation between Mycobacteriophage genomes.

**FIGURE 11:**
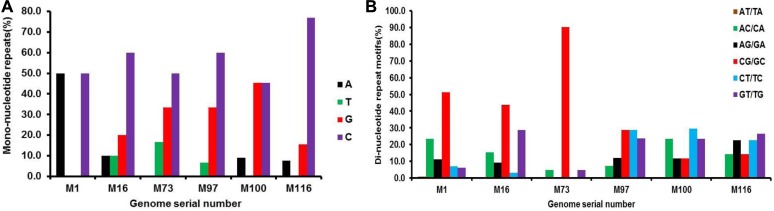
Composition of **(A)** mononucleotide and **(B)** dinucleotide repeat motifs in six Mycobacteriophage genomes selected by their host range. The broad host range species M97, M100, M116 have extremely high prevalence of mono-nucleotide repeat motifs G/C. These species have ∼20% of the di-nucleotide repeat motifs CT/TC, which are either absent or comparatively much less represented (<10%) in the others with narrow host range.

We also examined if SSR incidence was correlated with complexity regarding the host range of the Mycobacteriophages covered in the study. For this we focused on six phages: three with broad host ranges, *Mycobacterium phage L5* (M116), *Mycobacterium phage D29* (M100), and *Mycobacterium phage Bxz2* (M97); and three with restricted host ranges, *Mycobacterium phage barnyard* (M1), *Mycobacterium phage rosebush* (M73), and *Mycobacterium phage Che8* (M16) (Hatfull, 2014). We generated dot plots for these genomes using Gepard (Krumsiek et al., 2007) to highlight the presence of SSRs within the genomes, as represented in Figure 10. Though a repeat-rich genome is an apt platform for genomic evolution and diversity, it is dependent on constituent repeat motifs as well. We looked into the mono- and di-nucleotide repeats of these six genomes keeping in mind the host range. The three species with broad host range (M97, M100, M116) have around 90% of their mono-nucleotide repeat motifs composed of G or C. Also, except for M16, none of the species here has both A and T mononucleotide motifs (Figure 11A). Furthermore, around 20% of the di-nucleotide repeats motifs in the genomes exhibiting a broad host range were CT/TC, which were either absent or represented to a much lesser extent in the other genomes (Figure 11B). We did Pearson Chi-squared test and found that presence of different mono and dinucleotide repeat types were significant for broad host range and for restricted host range. The chi-square for mononucleotides was 48.75 (*P*-value <0.0001) and for dinucleotides 145.1 (*P*-value <0.0001).

## Discussion

Microsatellites or SSRs are present across prokaryotic and eukaryotic genomes. In this study, we screened 147 Mycobacteriophage genomes for the presence, abundance, and composition of SSR and cSSR tracts. Though a minor component of these variations may be attributed to sequencing errors but such artifacts, if at all present would be too miniscule to challenge the data *per se*. We find variation in the incidence of SSRs and cSSRs and their related parameters such as RA, RD, and cSSR percentage. We looked for correlations between frequency and composition of SSRs and cSSRs and found that type of motif varies in species with different host ranges. In our analysis, we found that dinucleotide repeats were most prevalent, followed by trinucleotide, mononucleotide, tetranucleotide, hexanucleotide, and pentanucleotide repeat motifs respectively. Repeat motifs were mostly present in the coding region. We found that genomes can differ in size by almost 16 kb but yet have almost the same number of SSRs. This variation might be associated with the ability to expand their host range through mutations in tail genes (Jacobs-Sera et al., 2012).

In the five Mycobacteriophage species examined, the cSSR percentage increases with increasing dMAX, but not linearly, as we saw earlier on *Flavivirus, Ebolavirus, Alphavirus, Human Papillomavirus (HPV), Potexvirus, Carlaviruses*, and *Tobamovirus* (Alam et al., 2013, 2014a,b,c; Singh et al., 2014; Mashhhood Alam, 2016). The non-linear conversion of SSRs to cSSRs in genomes of similar size suggests differential roles of repeat sequences. The cSSRs have been shown to be an outcome of recombination between homologous microsatellites (Jakupciak and Wells, 1999). Although imperfections in microsatellites have been proposed as a source of their evolution (Kofler et al., 2008), a clear evolutionary path of imperfect microsatellites in evolution is not yet elucidated. The abundance of microsatellites correlated well with the sequence composition of the repeat units. Poly (G/C) repeats were significantly more prevalent than poly (A/T) repeats in each complete Mycobacteriophage genome. This contrasts with most of eukaryotic and prokaryotic genomes, which have more abundant poly (A/T) tracts (Gur-Arie et al., 2000; Tóth et al., 2000; Karaoglu et al., 2005). In the sequences we analyzed, GC content is only slightly higher compared to AT, which suggests GC content has no influence on poly (G/C) repeats. Dinucleotide repeats were more prevalent compared to trinucleotide repeats. For the dinucleotide repeats, GC/CG predominates, whereas AT/TA was rare. Contrary to our results, CG/GC repeats were found rarely in most of earlier analyzed genomes such as geminivirus (George et al., 2012), human, *Drosophila* (Katti et al., 2001), *Arabidopsis thaliana, Oryza sativa, Triticum aestivum, Zea mays, Glycine max* (Morgante et al., 2002), fungi like *Aspergillus nidulans, Cryptococcus neoformans* (Hong et al., 2007), and some other eukaryotes (Deback et al., 2009). Among the trinucleotide repeats, GC-rich ones, such as GGC/CGG/AGC/CGA and GCG, were most prevalent.

In earlier studies it has been shown that each type of repeat sequence has its effects on genome function. The mononucletide repeat polymorphism can affect sporulation in budding yeast (Kashi and King, 2006). The dinucleotide repeats have been known to be associated with copy number variations, strand slippage, and polymorphisms accounting for genome evolution and adaptation (Tóth et al., 2000; Kashi and King, 2006; Deback et al., 2009). The contribution of dinucleotide SSRs motifs provides a platform for the dynamic nature of mycobacteriophage genomes, whereas trinucleotide repeats are present mostly in the coding region.

The analysis of L5like virus deserves a special mention across Figure 1, 3, 4. In Figure 4, which depict RA and RD there seems to be a discontinuity in graph for these species (M87 to M147). However, the same may be attributed to lower microsatellite incidence as clearly depicted in Figure 1. What makes it interesting is that in spite of lower values for incidence accounting for reduced RA and RD, these species have a very high cSSR% (Figure 3) implying the clustering of present SSRs. This further strengthens our understanding about SSRs being localized and implicated in functional genome evolution.

The range for RA and RD across genomes of mycobacteriophages is a representation of the degree to which microsatellites fill genome space; values of these parameters indicate species with the potential for genome evolution by SSR accumulation, reflecting on host divergence. Larger genomes tend to have more cSSRs, which suggests clustering of SSRs on the genome, because cSSR % is an indirect representation of distance between adjacent SSRs. Microsatellites are reportedly involved in regulation of gene expression and protein function in several species (Kashi and King, 2006; Chen et al., 2012). However, the fact that coding regions account for almost 78% of the total SSRs present is in line with our earlier studies (Alam et al., 2013, 2014a,b,c; Singh et al., 2014; Mashhhood Alam, 2016) across a diverse set of viruses. The actual presence of SSRs and cSSRs in the coding region and their effect on gene function may become clearer once all the gene products are properly studied and analyzed. We find a common trend that dinucleotide repeats were most prevalent followed by trinucleotide repeats in the coding region of various genera, which suggests their role in gene expression, regulation and evolution. Further, no similarities in the microsatellite landscape between viruses were observed in Herpesvirales having the same host (Wu et al., 2014a,b). It has been reported that the sum of individual incidences of mononucleotide repeat motifs doesn’t match the incidences of the corresponding di-nucleotide repeat motifs, which in turn contribute maximally to the SSR diversity in viral genomes (Zhao et al., 2012). The differences in repeat motif presence between those Mycobacteriophage having broad host range and those with restricted host range may be contributing to divergent host ranges by providing distinct platforms for each genome to evolve and diversify.

## Conclusion

The comparative genomics of phages that infect a single common bacterial host can help us understand the mechanisms giving rise to new viruses. The diversity in erstwhile Mycobacteriophages is probably an outcome of the ability of these viruses to rapidly adapt to new hosts. Genome-wide extraction of microsatellites across 147 Mycobacteriophage genomes revealed 25,284 SSRs and 1,127 compound SSRs (cSSRs). Interestingly, genomes of around 50kb accounted for similar numbers of SSRs as did a 110kb genome suggesting that SSR frequency is not necessarily a cause or effect of genome size. Also, a predominant localization of SSRs (∼78%) to coding regions when coupled to their established role in causing sequence polymorphisms indicates their pivotal role in functional genome evolution. Though a complete understanding of the proteins containing these SSRs is yet to be completed, the variations in motif constitution between species with different host range assign at least one functional role for these repeats. The broad host range species exhibited ∼90% mono-nucleotide repeat motifs representation of G/C and ∼20% of the di-nucleotide repeat motifs as CT/TC, which were either absent or represented to a much lesser extent in the other genomes.

## Author Contributions

CA and AS carried out the microsatellite extraction and correlation studies. AI developed the online tools. AHS helped in statistical analysis. SA coordinated the overall work and prepared the manuscript.

## Conflict of Interest Statement

CA was employed by Ingenious e-Brain Solutions, Gurugram, India. AI was employed by PIRO Technologies Private Limited, New Delhi, India. The remaining authors declare that the research was conducted in the absence of any commercial or financial relationships that could be construed as a potential conflict of interest.

## Footnotes

1 http://www.ncbi.nlm.nih.gov/genome/browse/

2 http://www.ncbi.nlm.nih.gov/

## Abbreviations

cSSR: compound simple sequence repeat
IMEx: imperfect microsatellite extraction
RA: relative abundance
RD: relative density
SSR: simple sequence repeat
